# Feasibility of Patient-Derived 3D Gastrointestinal Stromal Tumour Models as Alternatives for In Vivo Mouse Models

**DOI:** 10.3390/ijms262311456

**Published:** 2025-11-26

**Authors:** Dina Mönch, Julia Thiel, Meng Dong, Annika Maaß, Eileen Wegner, Anna Binner, Annette M. Staiger, Katrin S. Kurz, German Ott, Philipp Renner, Tobias Leibold, Christian Schmees, Thomas E. Mürdter, Matthias Schwab, Marc-H. Dahlke, Jana Koch

**Affiliations:** 1 Dr. Margarete Fischer-Bosch Institute of Clinical Pharmacology, 70376 Stuttgart, Germany; 2 University of Tübingen, 72074 Tübingen, Germany; 3 German Cancer Consortium (DKTK), German Cancer Research Center (DKFZ), 72076 Tübingen, Germany; 4 NMI Natural and Medical Sciences Institute, University of Tübingen, 72770 Reutlingen, Germany; 5 Department of Clinical Pathology, Robert-Bosch-Hospital, 70376 Stuttgart, Germany; 6 Department of General and Visceral Surgery, Robert-Bosch-Hospital, 70376 Stuttgart, Germany; 7 Department of Clinical Pharmacology, University Hospital Tübingen, 72076 Tübingen, Germany

**Keywords:** gastrointestinal stromal tumours, patient-derived 3D models, tumour microenvironment

## Abstract

Gastrointestinal stromal tumours (GISTs) are the most common mesenchymal tumours of the gastrointestinal tract and a key example for targeted therapy with tyrosine kinase inhibitors (TKIs), which have significantly improved survival rates. However, no effective treatments exist for TKI-resistant or mutation-negative tumours. Until now, research on the effects of TKIs has mainly used 2D cultures or mouse models, lacking patient-specific 3D GIST models. We investigated various 3D GIST models, including spheroids, organoids, patient-derived microtumours (PDMs), and precision-cut tumour slices (PCTSs), to assess their feasibility as alternatives for 2D cell culture or in vivo mouse models. Moreover, 2D monolayer and 3D spheroid GIST cell lines showed mutation-dependent responses to TKI treatment, but differences between 2D and 3D cultures were minimal. Thus, patient-derived 3D models, incorporating tumour microenvironment cells, were developed for more accurate in vivo representation. PDMs and PCTSs were successfully isolated from primary tumours and cultivated for up to two weeks. Three-dimensional models were immunohistochemically characterised, and the response to TKI therapies was tested and compared with expected clinical outcomes. In addition to already established 2D cell cultures and mouse models, PDMs and PCTSs are novel patient-derived 3D models that can be used to study tumour cell interactions within the microenvironment. Moreover, they could be used to investigate TKI resistance, and novel treatment options such as immunotherapies and combination therapies.

## 1. Introduction

Gastrointestinal stromal tumours (GISTs) are the most common mesenchymal tumours (“sarcoma”) of the gastrointestinal tract and, historically, a paramount example for targeted therapies with small molecule inhibitors. Approximately 85% of GISTs harbour activating mutations in the receptor tyrosine kinases *c-KIT* or *PDGFRA*. Less common driver mutations are found in the mitochondrial respiratory chain enzyme succinate-dehydrogenase, downstream signalling pathways (PI3K/AKT/mTOR-, JAK/STAT/JUN- or MAPK-pathway), or secondary tyrosine kinases [1,2].

Tumours with most of these mutations can be successfully treated with tyrosine kinase inhibitors (TKIs), which have proven to significantly prolong progression-free and median overall survival [3,4]. They can be used in neoadjuvant, adjuvant, and palliative settings. Although TKI-based therapies, such as imatinib, have revolutionised the overall survival of GIST-patients, they show only limited effect in patients with late-stage GISTs, escape mutations, or tumours with primary resistance. Secondary mutations in *c-KIT* or *PDGFRA* usually require the use of second- or third-line therapies [5,6,7,8]. However, progression-free survival upon treatment with these inhibitors is often only five to six months on average. In addition, therapy options for imatinib-resistant tumours or tumours without detectable driver mutations (“wild-type” GISTs) are still limited. Thus, current research focuses on novel TKI-based therapies, combination therapies with inhibitors of downstream signalling pathways, or immunotherapies [1]. The subgroup of patients who benefit most from these therapies still have to be determined.

Hence, from a clinical perspective, in vitro prediction of response to TKIs and other drugs is crucial in all of the above scenarios. Until now, the effects of TKI-based therapies and combination therapies in GIST have mostly been investigated in 2D cell cultures or murine in vivo models. This is mainly attributed to a lack of clinically relevant, patient-specific 3D GIST models, such as organoids or tissue slices, which have been widely used as response models for other malignancies, but not for sarcomas.

In this study, we established novel 3D GIST models that serve as a complement to already established 2D cell cultures and mouse models. These models can be used to investigate cellular interactions within the tumour microenvironment and to explore novel TKI-based therapies. Thus, we provide clinically relevant patient-derived GIST models that can be used in further patient-centered clinical investigations.

## 2. Results

### 2.1. 2D vs. 3D Cell Lines

GIST cell lines GIST-T1, GIST 48, and GIST 430 were analysed for their mutation status of *c-KIT* and *PDGFRA* as shown in Table 1. Based on their mutation status, GIST-T1 cells were predicted to be imatinib-sensitive, while GIST 48 and GIST 430 cells were considered imatinib-resistant [9].

**Figure 1:**
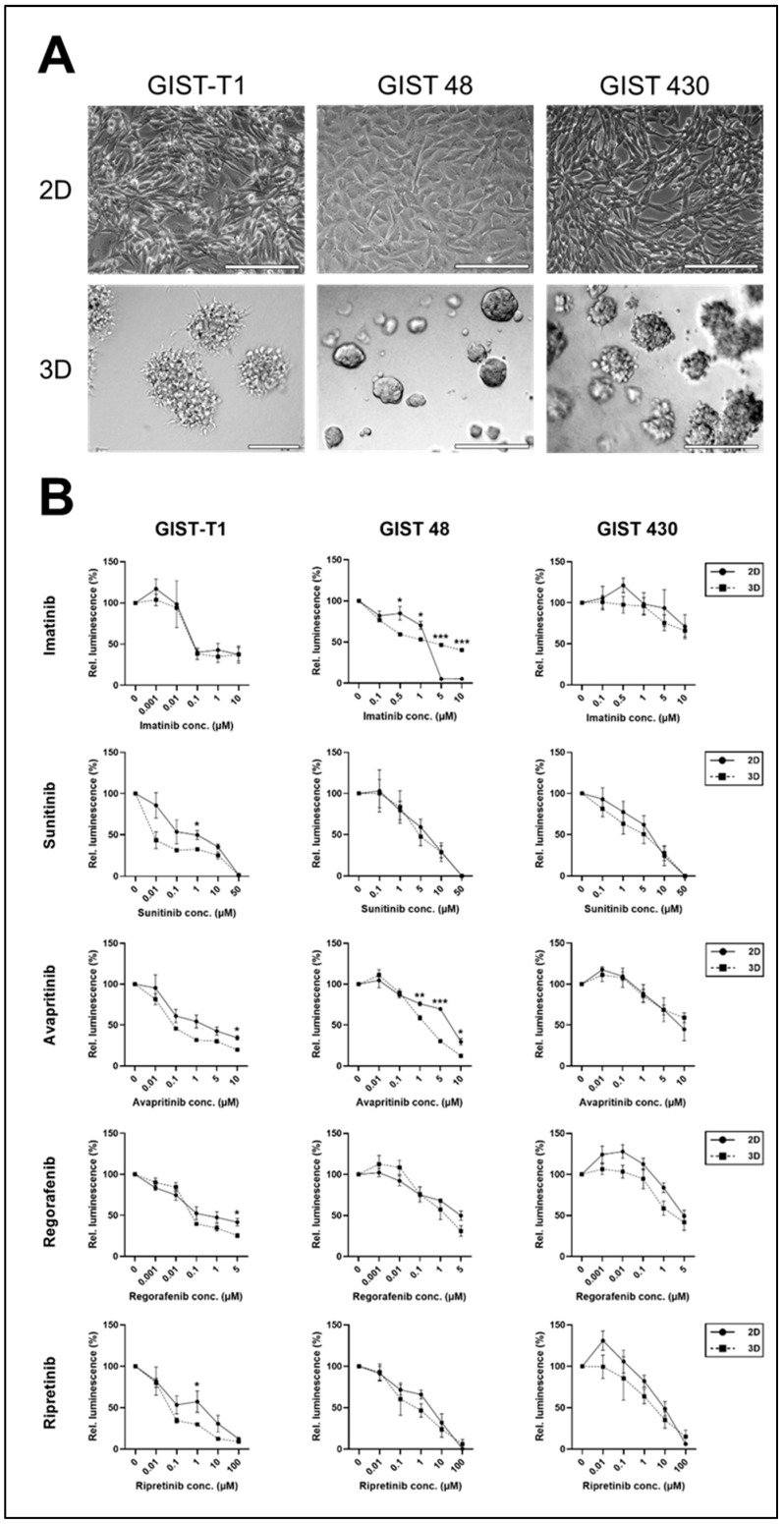
Response of 2D vs. 3D GIST cell lines to TKI treatment. (**A**) Microscopic images of 2D and 3D cultures of GIST cell lines; scale bars 200 µm. (**B**) TKI treatment of 2D (solid line) and 3D (dotted line) cultures at indicated concentrations (note that x-axes show ordinal values) for 72 h; mean and SE of at least three independent experiments; two-sided unpaired t-test; * *p* < 0.05, ** *p* < 0.01, *** *p* < 0.001.

Subsequently, GIST cell lines were grown either as 2D monolayers or 3D spheroid cultures. GIST-T1 and GIST 430 showed a grape-like growth pattern with irregular spheroid boundaries, whereas GIST 48 revealed a homogenous growth pattern with smooth membrane margins (Figure 1A). Standard-of-care treatment for GISTs includes imatinib and sunitinib as first- and second-line TKIs in patients with *c-KIT* mutations, as well as regorafenib and ripritinib as third-line TKIs. Avapritinib is usually prescribed for patients with *PDGFRA* mutations [1]. The response of 2D and 3D cultures to first-, second-, and third-line TKIs was analysed after 72 h in at least three independent experiments (Figure 1B). In general, GIST 48 and GIST 430 cells were more resistant to imatinib and sunitinib treatment in both culture systems compared to GIST-T1 cells. GIST 48 cells were significantly more resistant to imatinib treatment when cultivated as 3D spheroids and more resistant to avapritinib when cultivated as a 2D monolayer. GIST-T1 were more resistant in 2D culture when treated with sunitinib, avapritinib, regorafenib, or ripretinib. Lastly, 2D monolayer or 3D cultures of GIST 430 showed no significant differences in response to TKI treatments.

### 2.2. GIST Organoids

Organoids provide a more personalised approach than 3D spheroid cultivation of cell lines. Therefore, we next tested whether organoids could be isolated from GIST tissue. Organoid isolation was performed from GIST tissues of three patients. Isolated “organoids” were morphologically similar to a previously published case report of a GIST organoid [10] and could be kept in culture for up to three weeks, but they could not be passaged over more than two passages. Depending on the amount of tissue available, different media compositions and tissue dissociation methods were tested, but even in our most experienced hands and following stringent protocols, we could not establish GIST long-term cultures. We therefore considered organoids, at least following our current protocols, as a non-preferable in vitro model for further studies.

### 2.3. GIST Microtumours (PDMs)

**Figure 2:**
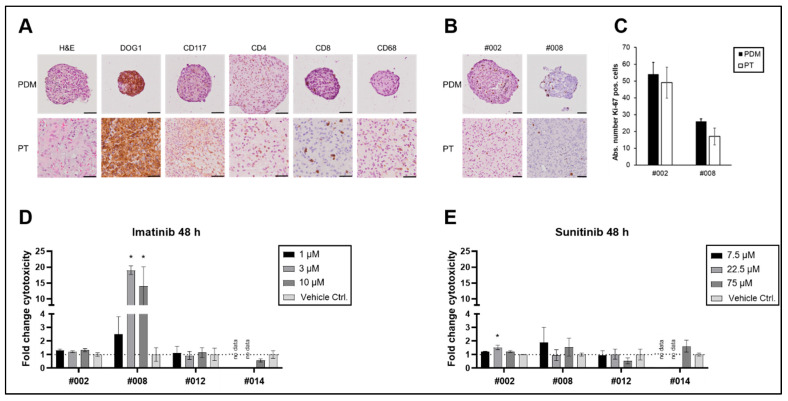
Characterisation of GIST PDMs and their response to TKI treatment. (**A**) IHC staining of patient-derived microtumours (PDMs) and corresponding primary tumour (PT); after isolation, PDMs were fixed, and FFPE sections were stained with markers for GIST (DOG1, CD117), T-helper cells (CD4), cytotoxic T-cells (CD8), and macrophages (CD68); scale bars 50 µm. (**B**) Ki-67 staining of PT and PDMs after isolation and (**C**) absolute number of Ki-67 positive cells in PDMs and PT; scale bars 50 µm. TKI treatment of PDMs with (**D**) imatinib or (**E**) sunitinib at indicated concentrations for 48 h; for easier comparison with control samples, dotted lines are shown at fold change cytotoxicity values of one; mean and SE of quadruplicates; two-way ANOVA with Dunnet’s multiple comparisons test, α = 0.05; * *p* < 0.05.

Thus, our further experiments establishing novel human ex vivo models focused on the establishment of PDMs from freshly isolated GIST samples directly after surgery. PDMs were isolated from four GIST patients (as previously described for other tumour types [11,12,13,14,15,16]) and characterised and compared with the corresponding primary tumour tissue specimens (Figure 2). IHC stainings revealed that GIST-typical tumour structures were conserved in PDMs with infiltration of immune and stromal cells. In addition, GIST and immune cell markers were comparable between PDMs and the corresponding primary tumour (Figure 2A).

Next, we used functional assays to assess the viability and proliferative capacities of PDMs as necessary prerequisites for further analyses, including the examination of therapy responses. Viability of the isolated PDMs was routinely assessed via live–dead cell staining, revealing stable viability of PDMs for up to 14 days (Supplementary Figure S1). The proliferative capacity of the PDMs was analysed by Ki-67 staining. As shown in Figure 2B,C, Ki-67 staining did not significantly differ between PDMs and the corresponding primary tumours.

To further elucidate whether PDMs are a suitable model system for assessment of drug treatment response in GIST, we analysed the effect of imatinib and sunitinib therapy on the viability of PDMs.

Sequencing analyses showed that three out of four patients harboured a *PDGFRA* or *c-KIT* mutation (Table 2). Whereas tumours without any driver-mutation (WT) or with *PDGFRA* mutations are expected to be imatinib-resistant, tumours with *c-KIT* mutations are in general imatinib-sensitive. PDMs of patient #002, who harboured a D842V mutation in exon 18 of PDFGRA and thus was expected to be imatinib-resistant, did not show any response to imatinib or sunitinib treatment (Figure 2D,E), which was in accordance with the clinical expectation (Table 2). In contrast, PDMs of patients #012 and #014, who were considered imatinib-sensitive according to their mutation profile, did not show any in vitro response (Figure 2D,E). PDMs of patient #008, whose tumour did not harbour any known driver mutation (WT GIST), showed an increase in cytotoxicity in response to increasing imatinib concentrations (Figure 2D). Although this contradicts the response expected based on the mutation profile, it is consistent with previous case reports that also show a response to treatment with imatinib in WT GIST [17]. At the same time, these PDMs were less sensitive to sunitinib treatment (Figure 2E). As far as clinical follow-up data were available, patients had no relapse for up to 27 months after surgery (Table 2). Due to the small sample size and the follow-up data that were not available for all patients, we were not able to draw any conclusions about the correlation between in vitro models and clinical follow-up data. Therefore, GIST PDMs need to be further validated in larger study cohorts. However, we still consider GIST PDMs a useful model system that allows for the investigation of interactions of tumour cells with cells of the tumour microenvironment in a clinically relevant setting.

### 2.4. GIST Slice Cultures (PCTS)

**Figure 3:**
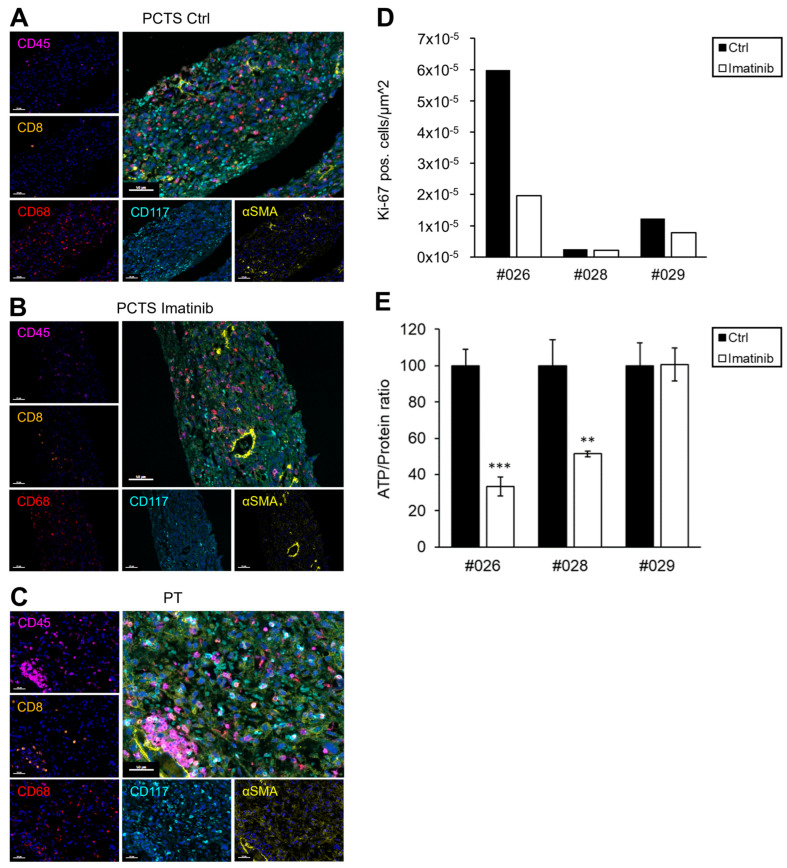
Establishment and characterisation of GIST slice cultures. (**A**–**C**) Multiplex immunofluo-rescence stainings of GIST slice cultures (PCTS) (**A**,**B**), and corresponding primary tumour (PT) (**C**). PCTS were treated with or without 2 µM imatinib for 5 days, and FFPE sections were stained with markers for haematopoietic blood cells (CD45), cytotoxic T-cells (CD8), macrophages (CD68), GIST (CD117), fibroblasts (αSMA), and nuclei (DAPI); scale bars 50 µm. (**D**,**E**) PCTSs were treated with 2 µM imatinib for 5 days. (**D**) The proliferation was analysed by Ki-67 staining, counting positive cells per µm^2^ using Qupath software. (**E**) Viability was measured via ATP assay and calculated in relation to protein mass relative to control treated samples; mean and SD of samples measured in triplicates; two-sided unpaired t-test relative to control, ** *p* < 0.01, *** *p* < 0.001.

We next tested whether GIST tissues from four patients could be cultivated as PCTS. Due to the solid structure and their low proliferative capacity, GISTs were easily sliceable and could be kept in culture for up to two weeks. In the following, GIST PCTSs from three patients were treated with 2 µM imatinib, and the response to TKI treatment was analysed via multiplex immunofluorescence (mIF) and Ki-67 staining and ATP-based viability assays (Figure 3). Multiplex staining of GIST PCTS and the corresponding primary tumour showed similarities with regard to immune- and stromal cell composition that were preserved during tissue cultivation (Figure 3A–C).

Sequencing analyses showed that all patients harboured either a *PDGFRA* or *c-KIT* mutation (Table 3). Thus, PCTSs from tumours with *c-KIT* mutations were considered imatinib-sensitive, while PCTSs from tumours with *PDGFRA* mutations were expected to be imatinib-resistant. PCTS of patient #026 responded to imatinib therapy at day 5, which was in accordance with clinical expectation (Figure 3D,E, Table 3). After 5 days of imatinib treatment, PCTS of patient #026 showed a 67% reduction in Ki-67 positive cells (Figure 3D) and a 67% reduction in ATP levels (Figure 3E). PCTS of patients #028 and #029 were expected to be imatinib-resistant and showed a 16% and 37% reduction in Ki-67 positive cells, respectively (Figure 3D). In addition, PCTS of patients #028 and #029 showed a 49% and 0% reduction in ATP levels, respectively (Figure 3E). Thus, 5 days after treatment, the PCTSs of patients #028 and #029 were considerably more resistant to imatinib treatment compared to #026, which was in accordance with clinical expectations. For prolonged treatment responses, we additionally measured ATP-levels of PCTS from patients #026-#029 at 10 and 14 days after treatment, as shown in Supplementary Figure S2. However, due to tissue limitations, measurements were not possible for all patients at all timepoints.

PCTSs from patient #024 were treated with lower imatinib concentrations and showed a reduction in Ki-67 positive cells and ATP-levels at day 5 (Supplementary Figure S3), which was in accordance with clinical expectations. Clinical follow-up data were available for two patients for up to 11 months and indicated no relapse (Table 3). Thus, the in vitro response of GIST PCTS correlated well with clinical expectations, and therefore, we consider them a useful model system with which to investigate therapy response and the interactions of tumour cells and cells of the tumour microenvironment in a clinically relevant personalised setting. However, like PDMs, GIST PCTSs need to be further validated in larger sample cohorts in order to better correlate the in vitro responses with clinical follow-up data.

## 3. Discussion

Over the last years, advanced 3D models such as organoids and tissue slices have contributed to a better understanding of tumour pathomechanisms and thus improved therapeutic approaches in many solid tumours. Due to a lack of personalised 3D models, preclinical research in GIST has relied, until now, mainly on established (2D) cell lines, such as GIST-T1, GIST 430, GIST 48 [19,20,21] or on (PDX) mouse models [22,23,24,25]. However, for personalised therapy approaches, clinically relevant, patient-specific 3D GIST models that integrate the tumour microenvironment are urgently needed.

Furthermore, rare subtypes that lack hallmark mutations in *c-KIT* or *PDGFRA* are underrepresented in the current landscape of GIST models, because their development has been proven time-consuming and technically difficult [26]. In this study, we tested the feasibility of different 3D GIST models such as spheroids from current GIST cell lines, as well as patient-derived organoids, PDMs and PCTSs. Especially, GIST PDMs and PCTS provide relevant model systems as they allow for the investigation of cell–cell interactions within the tumour microenvironment and probably also the therapy response of patient-derived GISTs in a personalised setting. However, the latter point needs to be further investigated in larger cohorts.

Here, we outline that the response of three different GIST cell lines to TKI treatment was dependent on their mutational status. This is well in-keeping with previous reports [9,27,28] and clinical experience [29,30]. In addition, we found that GIST-T1 cells were more sensitive to imatinib and sunitinib treatment when compared with GIST 48 and 430 cells. Moreover, GIST 48 cells were significantly more resistant to imatinib when cultivated as 3D spheroids and more resistant to avapritinib when cultivated in 2D. GIST-T1 and GIST 430 cells, however, did not show major differences in response to TKI treatment when cultivated as 2D monolayer or 3D spheroid cultures. This was in contrast to our expectations, as previous studies showed that 3D-cultured cell lines of other tumour entities are more resistant to chemotherapeutic and radiation treatment than 2D monolayers, which were attributed to cell–matrix and cell–cell interactions [31,32,33]. Gassl et al. also observed no difference between 2D and 3D cultures when comparing the chemosensitivity of pancreatic organoids in 3D to their corresponding 2D primary cell culture [34]. However, they transformed pancreatic 3D organoids to 2D monolayers in order to simplify the workflow and reduce the required overall cost and time. While this approach may be suitable for personalised prediction of patient chemotherapy responses, it might not be ideal for addressing more basic research-oriented questions. Fabro et al. observed heterogenous responses to TKI treatment between 2D and 3D in patient-derived glioblastoma cell lines and concluded that a mix of both systems should be used to comprehensively answer future research questions [35]. This highlights the importance of using models that more closely replicate the in vivo tumour environment, rather than relying solely on cell lines, for a comprehensive assessment of therapy response and resistance.

Since the first report of an organoid culture derived from human small intestine over a decade ago [36], organoid cultures have been established from a variety of tissues mimicking the key functional, structural, and biological complexity of their source material. Nowadays, their areas of application range from tissue regeneration, drug discovery, precision medicine approaches, and biomarker research to basic research [37]. In this study, we isolated organoids from three different GIST patients. Although the isolated material was morphologically similar to a previous published case report of a GIST organoid by Cao et al. [10], we did not see any proliferation or self-assembly over more than two passages. In our view, the isolated fragments more closely resembled enzymatically-digested tissue, capable of being maintained in culture for several days but not longer. A possible explanation for this could be that GISTs originate from differentiated Cajal cells of the gastrointestinal tract that are not cancer stem cells per se. Therefore, in fact, they do not provide the basis for true organoid assembly. Apart from Cao et al., the only other reports of successfully isolated GIST organoids were embedded in larger cohorts of human or canine gastrointestinal organoid biobanks [38,39]. None of them provides information on further experiments using these GIST organoids in long-term culture over several passages, which in our opinion is a key prerequisite for a successful organoid culture. All three isolated organoids were kept in a specific organoid medium based on the ones used in previous studies [10,12,38,39,40]. However, as the primarily used medium was based on the original organoid medium designed for stem cell-based tumours [36], we cannot exclude that successful GIST organoid culture would require additional or different media components.

In this study, we successfully established patient-derived PDMs and PCTS from GIST to investigate their suitability as novel 3D models. We found that GIST PDMs could be cultured with stable viability for up to two weeks and that their structure was comparable to the primary tumour with regard to immune and GIST-specific markers. In addition, Ki-67 staining did not differ between PDMs and the primary tumour. Upon treatment with imatinib, one out of four PDMs showed an in vitro response, with no PDM responding to sunitinib treatment. In particular, neither of the two PDMs with an imatinib-sensitive mutation in *c-KIT* exon 11 responded to TKI treatment after 48 h. Although PDM isolation, culture, and analysis have been successfully established for various tumour types, we cannot exclude that the chosen treatment duration was sufficient to achieve a response in slow-growing tumours such as GIST. In contrast, we observed a response in WT PDMs after 48 h of treatment with imatinib. Although WT GIST cases are known to have a significantly poorer response rate to imatinib [29,30], this cannot be excluded, as shown in a case report by Murray et al. [17]. In summary, the in vitro response matched the clinical therapy prediction based on the tumour mutation profile in one case.

Previous reports have shown that PCTS can be used to study the immune microenvironment and investigate the tumour response towards (immuno-) therapy [41]. We therefore tested whether PCTS could be generated from GIST tissue. PCTS were successfully generated from GIST and could be kept in culture for up to two weeks. Multiplex immunofluorescence stainings of PCTS and the corresponding primary tumours revealed similarities with regard to immune- and stromal cell composition during cultivation. In addition, the response to imatinib therapy was investigated in PCTS using Ki-67 stainings and ATP-based viability assays [42,43]. As expected, PCTSs from patient #026 were especially sensitive to imatinib treatment, while PCTSs from patients #028 and #029 that harboured a *PDGFRA* mutation were less sensitive to imatinib treatment 5 days after treatment. Thus, PCTS corresponded to the expected clinical response in all four cases, which is why we consider PCTS to be a valuable model with which to investigate patient response in a personalised setting.

PDMs and especially PCTS both provide useful model systems with which to investigate the interaction between tumour cells and cells of the tumour microenvironment such as fibroblasts and immune cells. Additionally, these model systems are probably well suited to studying the effect of TKI-based therapies and novel combination therapies in a more personalised setting, which has to be further tested and validated in larger cohorts. In this study, the response of PDMs to tested TKI treatments did not always match the clinical expectation based on their mutational profile. This might be attributed to the small sample cohorts studied here, which makes in-depth clinical prediction and correlation of in vitro results with clinical follow-up data impossible. Further limitations arose from the fact that GIST tumours are a rare tumour entity with a limited number of cases. In addition, in the case of small tumours, most of the surgical specimen is required for routine diagnostics and clinical molecular specification, leaving only a small portion available for research. As a result, only a limited number of experiments could be performed, and patient-material was sometimes so scarce that not all conditions could be tested simultaneously. Moreover, patient-specific influences of the tumour microenvironment could be responsible for the differences seen between the in vitro responses and clinical expectations. Various components of the tumour microenvironment, such as MDSCs, CAFs, or immune cells, were shown to have a strong influence on therapy response [44]. Thus, for better response prediction, it would be interesting to include cells of the immune system in our PDM and PCTS models, for example, via co-cultivation with TILs or PBMCs [11,12]. Based on our and previous studies [45], co-cultures including the tumour microenvironment in addition to the tumour mutation status must be considered for the development and analysis of novel GIST treatments, especially for immunotherapies which are the subject of current GIST research [46,47].

Moreover, the mutation status of GISTs was shown to influence the tumour microenvironment, as *PDGFRA*-mutant GISTs reveal a specific immune profile [48,49] that differs from GISTs with other mutations such as *c-KIT*. Furthermore, TKIs and especially imatinib can lead to changes in the immune cell population within the tumour [45,50] and thus affect the tumour microenvironment directly [51,52,53]. Consequently, immunotherapies are an interesting option for treatment of GIST, especially imatinib-resistant tumours. However, due to a lack of realistic patient-derived 3D models, the effect of immunotherapies and checkpoint inhibitors in combination with TKIs on the development of resistances is still unclear. For this, longitudinal experiments with patient-derived 3D models are needed in order to understand the development of resistance mechanisms to TKI-based therapies.

Different model systems come with their own strengths and limitations. It is well established that differences between human and murine cells, e.g., in growth factor or cytokine signalling, can lead to differences in growth behaviour, metastatic potential, and drug sensitivity [54,55]. The PDMs and PCTS models established here provide tools that have the potential to bridge the gap between the results obtained from 2D monolayer cell cultures and the reality in human solid tumours and furthermore reduce the amount of in vivo mouse experiments [54,56]. This approach is in line with the recent outline by the FDA that animal testing should be reduced in preclinical safety studies through scientifically validated new methods such as organ-on-a-chip systems, computational modelling, and advanced in vitro assays, which are highly relevant to human biology (FDA, Roadmap to Reducing Animal Testing in Preclinical Safety Studies, https://www.fda.gov/news-events/press-announcements/fda-announces-plan-phase-out-animal-testing-requirement-monoclonal-antibodies-and-other-drugs (2025), accessed on 5 August 2025). Moreover, a study by Roife et al. compared the therapy response of PDX tumourgrafts and corresponding tissue slices obtained from the PDX, showing that both in vivo and ex vivo tumours responded similarly to therapy with chemotherapeutics [57]. This further supports the use of ex vivo models to reduce animal experiments, costs, and valuable time when used for personalised medicine and therapy prediction.

## 4. Materials and Methods

### 4.1. GIST Cell Culture (2D and 3D)

GIST-T1 cells (PMC-GIST01C, Hölzel Diagnostika Handels GmbH, Cologne, Germany) were cultured in Advanced DMEM/F-12 medium (12634028, Fisher Scientific GmbH, Sindelfingen, Germany), containing 10% FBS (10270106, Fisher Scientific GmbH), 1% Penicillin/Streptomycin (L0022-100, VWR International, Radnor, PA, USA), and 1% L-Glutamine (11539876, Fisher Scientific GmbH). GIST 48, and GIST 430 [19] cell lines were a kind gift from Sebastian Bauer, University Hospital Essen, Germany.

GIST 48 and 430 cell lines were cultured in Iscove’s Modified Dulbecco’s Medium (IMDM, 12440053, Thermo Fisher Scientific, Waltham, MA, USA), containing 15% FBS, 1% Penicillin/Streptomycin, 1% Amphotericin, and 1% L-Glutamine, as established by the supplier (Sebastian Bauer, University Hospital Essen, Germany). GIST 430 cells were cultured with 200 nM of the TKI imatinib (STI571, SEL-S2475, Selleckchem LLC, Housten, TX, USA). Before use, mycoplasma contamination of cell lines was excluded by PCR analysis. All cell lines were cultured at 37 °C, 5% CO_2_ and splitted every one or two weeks when reaching 90% confluency.

For 2D assays, 10,000 cells per well were seeded in 96-well plates. For spheroid cultivation, 1250–5000 cells per well were seeded in 96-well plates, covered with 50 µL of 15% Matrigel^®^ (356231, Corning B.V., Amsterdam, The Netherlands), and diluted with the appropriate culture media. Cells were treated with TKI inhibitors three days after seeding for up to 72 h.

### 4.2. Organoid Culture

Patient-derived GIST organoids were generated and cultured as described earlier [40,58]. In brief, the tissue was cut into small pieces and dissociated at 37 °C. Dissociated cells were passed through a 30 and 100 μm cell strainer and cultured in either tumour organoid medium as described in detail in [10,40] and embedded in Matrigel^®^ (356231, Corning). For subcultivation, organoids were removed from Matrigel^®^, dissociated into small organoids using TrypLE (Thermo Fisher Scientific) and then transferred into fresh Matrigel^®^. For alternative approaches, tissue digestion was performed automatically with a GentleMACS octo dissociator (Miltenyi Biotec, Bergisch Gladbach, Germany) using the pre-installed “medium” tissue dissociation programme and the “human tumor dissociation mix” (Miltenyi Biotech) according to the manufacturer’s protocol. For subcultivation in an alternative medium, organoids were cultured in StemPro^®^ hESC SFM (Gibco, Waltham, MA, USA) supplemented with FGF-basic (Gibco), β-mercaptoethanol (Gibco), and Primocin (Invivogen, Waltham, MA, USA).

### 4.3. Generation and Processing of Patient-Derived Microtumours (PDMs) from Residual Fresh GIST Tissue

Freshly dissected GIST tissue was obtained during major surgery performed in the Department of General and Visceral Surgery at the Robert-Bosch-Hospital Stuttgart, Germany and maintained in DMEM/F12 culture media (Gibco) plus Primocin (Invitrogen) until it was subsequently processed as previously described [11,12,13,14,15,16]. Briefly, tumour tissue specimens were washed in HBSS (Gibco), fragmented with forceps, and digested with Liberase DH (Roche, Ludwigsburg, Germany) for 2 h at 37 °C. The digested tissue was filtered using cell strainers (Corning). Tumour fragments retained by cell strainers were washed in HBSS and cultured in suspension in StemPro^®^ hESC SFM (Gibco) supplemented with FGF-basic (Gibco), β-mercaptoethanol (Gibco), and Primocin (Invivogen) in a cell-repellent culture dish (60 × 15 mm) (Corning).

The viability of PDM was assessed by live/dead cell staining using Calcein-AM (Invitrogen) live cell stain and SYTOX™ Orange nucleic acid dead cell stain (Invitrogen, Waltham, MA, USA). After 30 min of incubation, z-stack images were taken using the Zeiss CellObserver Z1 (Carl Zeiss, Oberkochen, Germany) with 20× magnification.

For histology, PDMs were fixed in 4% Roti^®^ Histofix (Carl Roth, Karlsruhe, Germany) at RT and incubated for 5 min in Harris Hematoxylin (Leica Biosystems, Nussloch, Germany), shortly washed in dH_2_O, and dehydrated in an ethanol series (2× 50% EtOH, 2× 70% EtOH, 15 min each). Using Tissue-Tek^®^ Cryomolds^®^ (Sakura, Torrance, CA, USA), PDMs were embedded in Richard-Allan Scientific™ HistoGel™ (Thermo Fisher Scientific). Further tissue processing was performed as described before [10,11].

### 4.4. Precision-Cut Tumour Slice (PCTS) Generation

PCTS generation of patient-derived GIST tissue was performed as described earlier [56]. Tumour tissue slices were prepared at a thickness of 250 μm using the Leica VT1200S vibrating blade microtome (Wetzlar, Germany). Tissue slices were maintained on a Millipore filter (Millicell Cell Culture inserts, Merck Millipore, Burlington, MA, USA, PTFE, pore size 0.4 μm) and cultured at 37 °C and 5% CO_2_ in a humidified atmosphere under atmospheric oxygen (21% oxygen) conditions. PCTS were cultured in advanced DMEM/F-12 medium (12634028, Fisher Scientific GmbH), containing 10% FBS (10270106, Fisher Scientific GmbH), 1% Penicillin/Streptomycin (L0022-100, VWR International), and 1% L-Glutamine (11539876, Fisher Scientific GmbH).

The tissue fixed immediately after surgical resection was defined as the in vivo sample. The tissue after the slicing process and before tissue slices cultivation was defined as the d0 sample. Fixed slices were embedded in paraffin in a vertical orientation as published in Davies et al., 2015 [56].

### 4.5. TKI Treatment

Small molecule inhibitors imatinib (STI571, SEL-S2475, Selleckchem LLC), sunitinib (HY-10255A, Med-ChemExpress, Monmouth Junctions, NJ, USA), avapritinib (HY-101561, MedChemExpress, Monmouth Junction, NJ, USA), ripretinib (A19508, Adooq Bioscience LLC, Irvine, CA, USA), and regorafenib (A10250, Adooq Bioscience LLC) were dissolved in DMSO as 1 mM stock solutions. Working solutions were freshly prepared and diluted in culture medium.

### 4.6. Viability Assays

Cell viability of 2D and 3D cell cultures and PDMs was assessed using a RealTime-Glo™ MT Cell Viability Assay (G9712, Promega, Madison, WI, USA) according to the manufacturer’s instructions. For each PDM treatment, three to five replicates each with n = 15 PDMs were prepared in phenol-red free culture medium with a total volume of 150 µL.

Tissue lysates of PCTS were prepared in 2 mM EDTA in 70% EtOH (*v*/*v*) using lysing matrix D tubes with the FastPrep sample preparation system (MP Biomedicals, Santa Ana, CA, USA). The resulting supernatant was used to assess the viability of PCTSs using an ATP Bioluminescence-Assay (11699695001, Merck Chemicals GmbH, Darmstadt, Germany) according to the manufacturer’s instructions. The ATP values were normalised to the protein content of each sample. The precipitate of the PCTS tissue lysates were dried overnight. Proteins were lysed in 5 N NaOH and homogenised using the FastPrep sample preparation system. Protein measurement was performed with Pierce BCA Protein Assay Kit (23227, Thermo Fisher Scientific).

### 4.7. Immunohistochemical (IHC) Staining

FFPE sections of 4 μm thickness were stained with Mayer’s hematoxylin (Sigma-Aldrich Chemie, St. Louis, MO, USA) and eosin (Merck Chemicals GmbH). IHC was carried out by standard protocols using the Dako Envision Kit (Dako, Glostrup, Denmark) according to the manufacturer’s manual. For CD117 (Clone YR145, 1:200, Cell Marque, Rocklin, CA, USA), CD4 (Clone SP35, 1:100, Cell Marque) and CD68 (Clone Kp-1, 1:8000, Cell Marque), antigen retrieval was performed with citric acid buffer pH 6. For DOG1 (Clone SP31, 1:50, Cell Marque), CD8 (Clone SP16, 1:100, Cell Marque), and Ki-67 (Clone SP6, 1:75, Cell Marque) antigen retrieval was performed with Tris/EDTA buffer pH 9 (Dako, Glostrup, Denmark). Primary antibodies were incubated for 30 min.

### 4.8. Quantification of Proliferation

Tumour cell proliferation was quantified by the expression of Ki-67 protein, which is produced during cell division. Ki-67 staining was performed as standard as described above. Only nuclear Ki-67 immunoreactivity was considered positive. Cells were counted using 40× magnification, counting at least 436 cells per tumour. Alternatively, the proliferation was analysed by Ki-67 staining, counting positive cells per µm^2^ using Qupath software 0.6.0.

### 4.9. Multiplex Immunofluorescence (mIF) Staining

A six-colour multiplex immunofluorescence staining was performed using the tyramide signal amplification-based OPAL^TM^ multiplexing method. The staining protocol for FFPE tissue sections was optimised for simultaneous detection of 5 antibodies. Nuclei were stained with DAPI. FFPE-fixed tissues were cut into 3 µm sections using a rotary microtome (Leica R2255, Leica Biosystems). Sections were deparaffinised, rehydrated, subjected to heat-induced epitope retrieval, and incubated with primary and secondary antibodies. Antibodies were visualised with fluorescent tyramides of the Opal 6-Plex Manual Detection Kit (NEL861001KT, Akoya Biosciences, Marlborough, MA, USA). The process of epitope retrieval at pH 6 and staining for 30 min was repeated sequentially for different primary antibody and fluorescent tyramide combinations. The following primary antibodies were used: αSMA (ab5694, 1:100, Abcam, Cambridge, UK), CD8 (Clone SP16, #108R, 1:100, Cell Marque Corporation), CD68 (Clone Kp-1, #168M, 1:300, Cell Marque), CD117 (Clone YR145, #117R, 1:200, Cell Marque) and CD45 (Clone D9M8I, #13917, 1:100, Cell Signaling Technology, Danvers, MA, USA). Antibodies were visualised with the following tyramide fluorophores: Opal Polaris 480, Opal 520, Opal 570, Opal 620, Opal 690 and Opal 780. Stained sections were mounted using the ProLong Diamond Antifade Mountant (P36961, Thermo Fischer Scientific) and imaged using the PhenoImager Fusion system (Akoya Biosciences).

### 4.10. Mutation Analysis

Sanger sequencing was performed for gene mutation analysis of common GIST mutations in *c-KIT* exon 9 and 11 and *PDGFRA* exon 18. DNA from fresh tissue was isolated using the DNeasy Blood and Tissue Kit (69504, Qiagen, Hilden, Germany) according to the manufacturer’s instruction. DNA from cell pellets or FFPE tissue was extracted using the AllPrep DNA/RNA FFPE Kit (Qiagen) according to the manufacturers protocol. The PCR master mix was prepared using 200 ng DNA, 10× PCR-Puffer (Qiagen), dNTPs (1,25 mM each), Hot Star Taq (5 U/µL, Qiagen), and forwards and reverse primers (10 pmol/µL each). Initial denaturation was performed for 15 min at 95 °C, followed by 35 cycles of amplification, each consisting of denaturation for 30 s at 95 °C, annealing for 30 s at 58 °C, and elongation for 30 s at 72 °C. Final elongation was completed by 10 min at 72 °C. PCR was performed using the primers *c-KIT* e9for 5′TCCTAGAGTAAGCCAGGGCTT, *c-KIT* e9rev 5′TGGTAGACAGAGCCTAAACATCC; *c-KIT* e11for 5′CCAGAGTGCTCTAATGACTG, *c-KIT* e11rev 5′AGCCCCTGTTTCATACTGAC; *c-KIT* e13for 5′CATCAGTTTGCCAGTTGTGC, *c-KIT* e13rev 5′AATCTAGCATTGCCAAAATCA; *c-KIT* e17for 5′TTTCTCCTCCAACCTAATAG, *c-KIT* e17rev 5′CCTTTGCAGGACTGTCAAGC; *PDGFRA* e12for 5′TCCAGTCACTGTGCTGCTT, *PDGFRA* e12rev 5′GGGAGTCTTGGGAGGTTACC; *PDGFRA* e18for 5′AGTGTGTCCACCGTGATCTG, *PDGFRA* e18rev 5′TGAAGGAGGATGAGCCTGACC. The preamplification PCR product was controlled by gel electrophoresis and purification was conducted with DNA Clean and Concentrator from Zymo according to the manufacturers’ instructions (Zymo research, Freiburg, Germany). Sanger sequencing PCR of purification products was carried out using the Big Dye Terminator v3.1 Cycle Sequencing Kit (Applied Biosystems, Foster City, CA, USA). Sequencing PCR products were purified using a Sephadex column and samples were analysed on a 3500Dx Genetic Analyzer (Applied Biosystems). Samples were compared to a wild-type reference sequence (*KIT* NM_000222.2, available under https://www.ncbi.nlm.nih.gov/clinvar/RCV000458760.3/, accessed on 5 August 2025, *PDGFRA* NM_006206.5, available under https://www.ncbi.nlm.nih.gov/clinvar/33687042/, accessed on 5 August 2025) using Chromas Lite 2.01 Software (Technelysium Pty Ltd., South Brisbane, Australia, 2007).

### 4.11. Statistical Analysis

Statistical analysis was performed with GraphPad Prism Version 10 (GraphPad Software Inc., San Diego, CA, USA). Treated samples from cell culture experiments were compared to control groups, and the *p*-values were calculated using either ordinary two-way ANOVA with Dunnet’s multiple comparisons test (mean and SE of quadruplicates, α = 0.05) or two-sided unpaired t-tests (mean and SD/SE of triplicates/three independent experiments as indicated in the figure legends). *p*-values were indicated as follows: *p* < 0.05 (*), *p* < 0.01 (**), and *p* < 0.001 (***).

## 5. Conclusions

In summary, we showed that patient-derived model systems, such as PDMs and PCTS, are feasible 3D models for GIST, enabling the investigation of interactions between tumour cells and their tumour microenvironment. These model systems need additional (clinical) validation and could be further improved, but we still consider them suitable model systems with which to understand the development of resistance mechanisms to TKI-based therapies and to explore novel GIST treatments such as immunotherapies and combination therapies.

## Figures and Tables

**Table 1:** Mutational characteristics of GIST cell lines.

Cell Line	*c-KIT*				*PDGFRA*	
	Exon 9	Exon 11	Exon 13	Exon 17	Exon 18	Exon 12
GIST-T1	WT	V560_Y578del	K642E (homozygous)	WT	WT	WT
GIST 48 *	WT	V560D (homozygous)	WT	D820A	WT	WT
GIST 430 *	WT	V560_L576del	V654A	WT	WT	WT

* imatinib-resistant, WT: wild-type.

**Table 2:** Clinical characteristics of PDMs and correlation of the in vitro response with clinical expectation.

Pat ID	Mutation Status	Ki-67 Index	Risk Stratification (Miettinen [18])	Expected Clinical Response	TKI-Therapy After Resection	PDM Response	Match In Vitro vs. Expectation	Clinical Follow-Up (Months After Surgery)
#002	*PDGFRA* exon 18: D842V	-	very low	imatinib-resistant	no TKI therapy recommended	-	yes	no relapse (27)
#008	WT	<1%	intermediate	imatinib-resistant	no TKI therapy recommended	++	no, but case reports with successful therapy [17]	-
#012	*c-KIT* exon 11: V559D	-	low (3a)	imatinib-sensitive	patient decision: no TKI therapy	-	no	no relapse (26)
#014	*c-KIT* exon 11: W557R	10%	low (3a)	imatinib-sensitive	individual decision: no TKI therapy	-	no	-

-: no response, ++: response at >one time point and/or concentration.

**Table 3:** Clinical characteristics of PCTS and correlation of the in vitro response with clinical expectation.

Pat ID	Mutation Status	Ki-67 Index	Risk Stratification (Miettinen [18])	Expected Clinical Response	TKI-Therapy After Resection	PCTS Response (Ki-67/ATP Assay)	Match In Vitro vs. Expectation	Clinical Follow-Up (Months After Surgery)
#024	*c-KIT* exon 11: 1648-4_1673del	<1%	-	imatinib-sensitive	permanent imatinib treatment	17%/38% (due to lower concentration)	yes	no relapse (16)
#026	*c-KIT* exon 9: A502_Y503dup	<5%	-	imatinib-sensitive	no TKI therapy recommended	67%/67%	yes	-
#028	*PDGFRA* exon 18: D842V	3–4%	3b	imatinib-resistant	no TKI therapy recommended	16%/49%	yes	no relapse (7)
#029	*PDGFRA* exon 18: D842V	<5%	2	imatinib-resistant	no TKI therapy recommended	37%/0%	yes	-

## Data Availability

The original contributions presented in this study are included in the article and Supplementary Materials. Further inquiries can be directed to the corresponding author.

## Supplementary Materials

The following supporting information can be downloaded at: https://www.mdpi.com/article/10.3390/ijms262311456/s1.

## Abbreviations

GIST	gastrointestinal stromal tumours
TKI	tyrosine kinase inhibitors
PDM	patient-derived microtumours
PCTS	precision-cut tumour slices
PDGFRA	platelet-derived growth factor
