# Supplementary material for: Feasibility of Patient-Derived 3D Gastrointestinal Stromal Tumour Models as Alternatives for In Vivo Mouse Models
Source: Int J Mol Sci. 2025 Nov 26;26(23):11456. doi: 10.3390/ijms262311456 (PMC12691747; doi:10.3390/ijms262311456)
Supplement: Supplementary file 1 [file ijms-26-11456-s001.zip › ijms-3960924-supplementary.pdf]

Supplementary

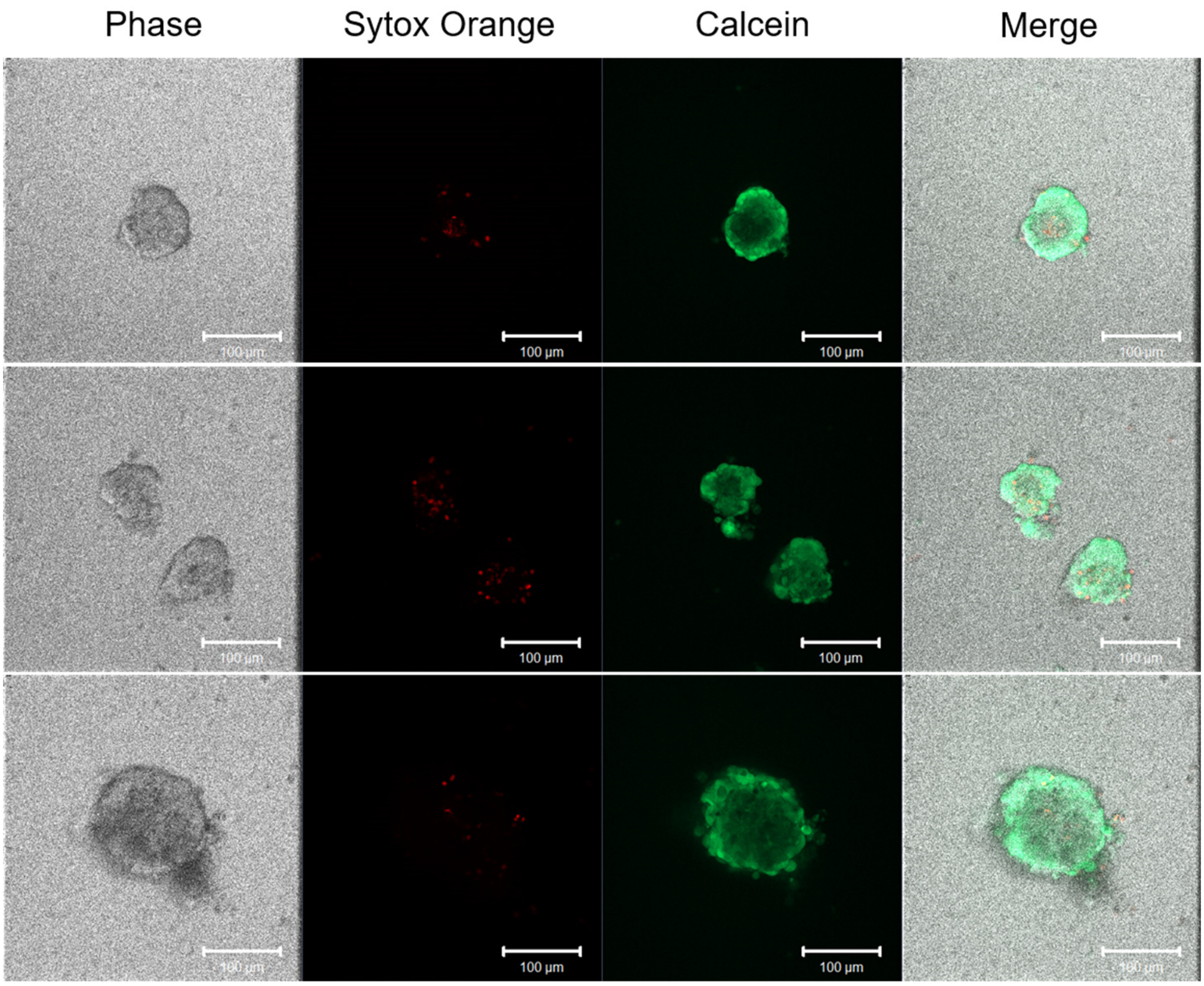

Supplementary Figure S1: Viability of GIST PDMs. Viability of PDMs was assessed by live/dead-cell staining using Calcein-AM live cell stain and SYTOX™ Orange nucleic acid dead cell stain. After 30 min of incubation, z-stack images were taken using the Zeiss CellObserver Z1 with 20x magnification; scale bars 100 µm.

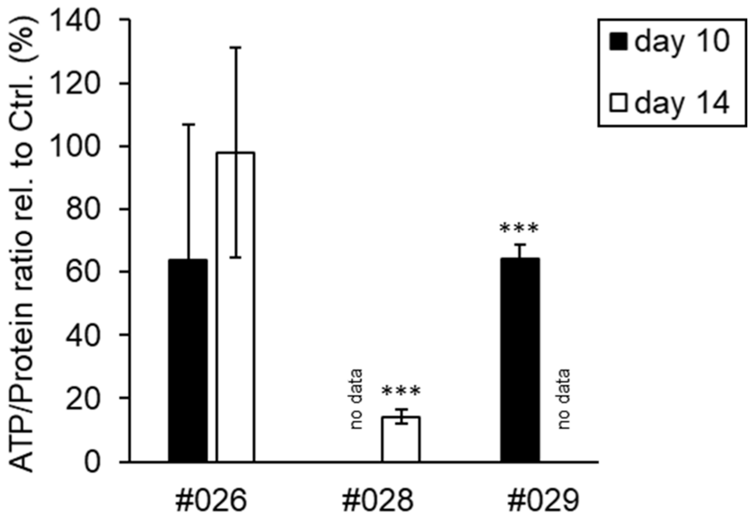

Supplementary Figure S2: Response to prolonged imatinib treatment. PCTS were treated with 2 µM imatinib for 10 or 14 days and viability was measured via ATP-assay and calculated in relation to protein mass relative to control treated samples; mean and SD of samples measured in triplicates; two-sided unpaired t-test relative to control, *** p<0.001.

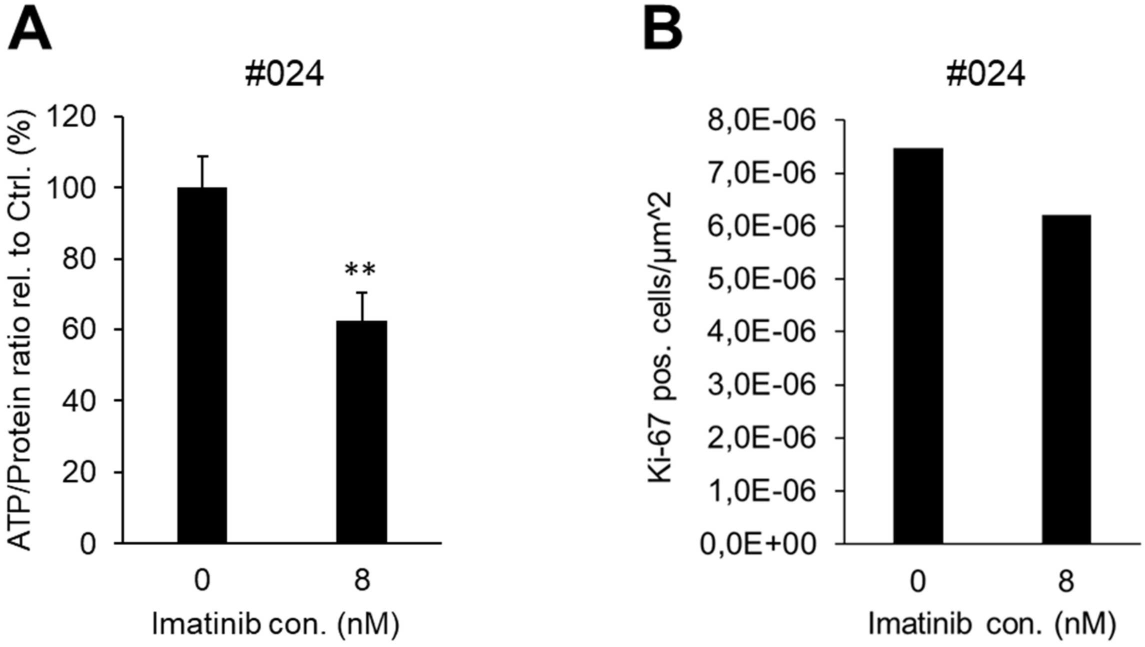

Supplementary Figure S3: Imatinib response of PCTS #024. A), **B)** PCTS were treated with 8 nM imatinib for 5 days. **A)** Viability was measured via ATP-assay and calculated in relation to protein mass relative to control treated samples; mean and SD of samples measured in triplicates; two-sided unpaired t-test relative to control, ** $p<0.01$. **B)** The proliferation was analysed by Ki-67 staining, counting positive cells per µm^2 using Qupath software.
